# A Simpler Machine Learning Model for Acute Kidney Injury Risk Stratification in Hospitalized Patients

**DOI:** 10.3390/jcm11195688

**Published:** 2022-09-26

**Authors:** Yirui Hu, Kunpeng Liu, Kevin Ho, David Riviello, Jason Brown, Alex R. Chang, Gurmukteshwar Singh, H. Lester Kirchner

**Affiliations:** 1 Department of Population Health Sciences, Geisinger Health, Danville, PA 17822, USA; 2 Department of Computer Science, Portland State University, Portland, OR 97201, USA; 3 Fresenius Medical Care North America, Waltham, MA 02451, USA; 4 Anticipatory Management Program, Steele Institute, Geisinger Health, Danville, PA 17822, USA; 5 Phenomics Analytics and Clinical Data Core, Geisinger Health, Danville, PA 17822, USA; 6 Department of Nephrology, Geisinger Health, Danville, PA 17822, USA

**Keywords:** hospitalization-acquired acute kidney injury, early detection, predictive modeling, machine learning, prediction, model, AKI, acute kidney injury

## Abstract

Background: Hospitalization-associated acute kidney injury (AKI), affecting one-in-five inpatients, is associated with increased mortality and major adverse cardiac/kidney endpoints. Early AKI risk stratification may enable closer monitoring and prevention. Given the complexity and resource utilization of existing machine learning models, we aimed to develop a simpler prediction model. Methods: Models were trained and validated to predict risk of AKI using electronic health record (EHR) data available at 24 h of inpatient admission. Input variables included demographics, laboratory values, medications, and comorbidities. Missing values were imputed using multiple imputation by chained equations. Results: 26,410 of 209,300 (12.6%) inpatients developed AKI during admission between 13 July 2012 and 11 July 2018. The area under the receiver operating characteristic curve (AUROC) was 0.86 for Random Forest and 0.85 for LASSO. Based on Youden’s Index, a probability cutoff of >0.15 provided sensitivity and specificity of 0.80 and 0.79, respectively. AKI risk could be successfully predicted in 91% patients who required dialysis. The model predicted AKI an average of 2.3 days before it developed. Conclusions: The proposed simpler machine learning model utilizing data available at 24 h of admission is promising for early AKI risk stratification. It requires external validation and evaluation of effects of risk prediction on clinician behavior and patient outcomes.

## 1. Introduction

Acute Kidney Injury (AKI) is a complex pathophysiological entity with various causes culminating in a sudden decrease in kidney function. Reported in up to one in five hospitalized patients, the occurrence of AKI has widespread adverse effects on clinical outcomes as well as economic costs [1]. In a worldwide meta-analysis representing close to 50 million patients, the pooled incidence rate of AKI was even higher in North America: close to 25%. Patients with AKI were at almost five times the risk of death compared to patients without AKI. This risk increases with increasing severity of AKI, going up to 25-fold among AKI patients requiring dialysis [2]. AKI has also been associated with an average of 3.5 days longer hospital lengths of stay and an additional $7500 in healthcare expenditure per patient [3]. Survivors of AKI continue to struggle with increased risks of residual disability, non-recovery of kidney function, worsening chronic kidney disease (CKD) and development of end-stage kidney disease (ESKD) [1].

The Kidney Disease Improving Global Outcomes (KDIGO) AKI workgroup clinical practice guidelines identified standard ways of defining AKI and recommended further research and evaluation of methods to predict risk of developing AKI [4]. Early AKI prediction and risk stratification holds promise to improve several steps in care: preventive interventions, closer monitoring, early diagnosis and institution of bundled interventions and/or clinical decision support systems [5]. Several predictive models have attempted early AKI risk stratification in hospitalized patients. Unfortunately, many have used non-standardized AKI definitions, suboptimal methodology, or were specific to particular sub-populations, hence limiting their utility [6].

More recently, advances in clinical informatics and availability of electronic health data have led to development of predictive algorithms utilizing artificial intelligence and machine learning. A key characteristic of these algorithms is the ability to incorporate multiple data-points from electronic health records (EHR) into AKI risk stratification [7]. Most of these models utilize supervised machine learning classification models, with some utilizing deep learning and recurrent neural networks. Many of these models use continuous data input to run ongoing predictions to improve performance [8]. More complex algorithms add to computational resource use, costs of implementation, and limit the clinical application of prediction models. Higher number of variables and disparate data use also predisposes prediction models to issues such as overfitting and hyperparameters, further limiting usability and generalization [8,9].

While existing AKI prediction models offer good predictive capabilities, they utilize several dozen variables to make an AKI prediction. When applying these models for practical use in EHR, there remain concerns that data input disruption or fluctuation in data quality in any one of the variables can disrupt the entire model output. For instance, authors from the Yale School of Medicine have described a significant disruption in an AKI alert system simply because the serum creatinine field was renamed in the EHR. Adjusting model inputs in response to such occurrences warrants simpler model design. Moreover, complex models yield a relatively high proportion of false positive predictions [10]. Given these valid concerns, we aimed to develop a simpler AKI prediction model. Our objective was to limit the numbers of variables used to accurately predict AKI by using the 20 strongest predictors obtained during the first 24 h of hospitalization. The 24 h window was chosen to reduce the computational burden required to run the model and informed by prior successful attempts that were limited in methodology or sub-populations studied [11,12].

## 2. Materials and Methods

### 2.1. Setting and Participants

Geisinger Health is an integrated healthcare system providing care to more than 3 million residents in and around central Pennsylvania. The system is comprised of 10 acute care hospitals, 2 research centers, a medical school, an insurance company, >1700 physician group practice and >900 advanced practitioners. All inpatient admissions in this system from 13 July 2012–11 July 2018 who had a serum creatinine measured were included in this study. Patients with ESKD, kidney transplant, length of stay less than 48 h and AKI at admission were excluded.

### 2.2. Definitions of AKI and Baseline Kidney Function

AKI was defined according to changes in serum creatinine as specified in KDIGO guidelines [4]. To reduce sample contamination by very transient AKI and lab error, patients were classified as having AKI if rise in creatinine met at least KDIGO stage 1 AKI criteria for a minimum of 48 h [13]. The baseline creatinine used to adjudicate AKI was determined using a validated hierarchy, utilizing a lookback period ranging between 14 to 365 days prior to admission as the first preference. This definition was chosen in view of a wealth of AKI phenotyping literature since the KDIGO guidelines were released [14]. This hierarchy is represented in Table 1.

### 2.3. Predictors

#### Predictors and Missing Data

To identify relevant candidate predictors, the full texts of 53 previously published hospitalization-associated AKI prediction models in various sub-populations were reviewed by at least 2 authors (at least one of the two had to be a nephrologist). A list of 746 candidate variables studied in these models was compiled [6]. These lists were presented and reviewed during our research group meetings to establish consensus on variables of interest. Any variables appearing in more than one predictive model previously were considered. Clinicians also suggested a few new variables of interest (example: antidiabetic medications, steroid use).

Variables measuring similar parameters but named differently were grouped together (example: essential hypertension, history of hypertension, high blood pressure, BP > 140/90, systolic hypertension, and diastolic hypertension were all grouped under hypertension). With the help of clinical pharmacists, relevant medications were grouped together into groups of interest using the American Hospital Formulary Service Pharmacologic-Therapeutic Classification System. Examples of medication groups of interest include angiotensin-converting-enzyme inhibitors/angiotensin receptor blockers (ACE/ARB), anticoagulants, antiplatelet agents, nonsteroidal anti-inflammatory drugs (NSAIDs), immunosuppressants, etc. At the end of this curation process, 121 variables were tested for inclusion into the model. A list of the variables tested is available in the Supplemental Table S1.

Variables of interest were then collected from EHR data including demographics, comorbid conditions, medication use, vital signs, surgical details (if pertinent) and laboratory values taken within the first 24 h of admission. Estimated glomerular filtration rate (eGFR) was calculated by using the Chronic Kidney Disease Epidemiology Collaboration (CKD-EPI) equation [15]. Laboratory values were ranked by total amount of missingness and split into common measures (whose missing rate was less than 50%) and rare measures (missing in >50% encounters). Multiple imputation by chained equations (MICE) was implemented for common variables [16]. Variables with missing rates greater than 85% after imputation were eliminated from the prediction algorithms.

### 2.4. Machine Learning Algorithms

Hospitalizations were divided into 2 groups: a training cohort comprised of admissions from 2012 through 2017 and a hold-out cohort comprised of admissions through 2018. Machine learning algorithms were implemented using variables of interest available in the first 24 h of admission to predict encounters where the patient developed AKI during the hospitalization. Predictive algorithms included penalized logistic regression using least absolute shrinkage and selection operator (LASSO), bootstrap aggregating (random forest) and boosting (gradient boosting machines). The optimal hyperparameters were selected by random search to optimize the model performance from 3-fold cross-validation in the training cohort.

Logistic regression is a simple and efficient classification algorithm, where each predictor’s importance is explicitly denoted by its corresponding coefficient. LASSO is a L1 penalized regression approach which attempts to avoid overfitting by forcing the coefficients of the least contributive predictors to be exactly zero [17]. Random forest is a machine learning algorithm that trains a number of decision trees using a combination of bootstrap aggregating and random feature selection. The hyperparameter set being tuned includes the number of trees, tree size, and number of features to consider per split in random forest. Random forest can prevent overfitting by creating random subsets of the features and training data for each decision tree [18]. Gradient boosting machines is a machine learning algorithm that converts several weak leaners into a strong learner by correcting its predecessor’s mistakes in a sequential manner. The hyperparameter set being tuned includes the number of trees to construct, the maximum depth of each tree and the learning rate. Early stopping can avoid overfitting by finding the least number of iterations that is sufficient to build the model and generalizes well to unseen data [19].

### 2.5. Performance Evaluation and Statistical Analysis

Candidate models were selected based on the result of 3-fold cross validation. The selected model was retrained using the whole training cohort and evaluated using the hold-out cohort. Algorithm performance were evaluated in the hold-out cohort using area under the receiver operating characteristic curve (AUROC) and precision-recall curves for separability for encounters with and without AKI, and trade-off between precision and recall for different thresholds, respectively. The probability cut-off point for the AKI prediction outcome was determined based on Youden’s Index to maximize the differentiating ability when equal weight is given to sensitivity and specificity. Model performance was then evaluated via 2 by 2 confusion matrix and comparing the sensitivity vs. specificity, AUROC, and negative predictive value (NPV) based on the confusion matrix. Statistical analyses were performed using R Statistical Software (RStudio Version 1.0.153).

## 3. Results

### 3.1. Baseline Characteristics

After meeting the prespecified inclusion and exclusion criteria, 209,300 unique admissions comprised our final cohort. Out of these, 26,410 (12.6%) had developed AKI associated with their hospitalization. Table 2 shows the clinical characteristics of those with and without AKI. Patients who developed AKI were older (70 vs. 63 years; *p* < 0.001), and more likely to be male (52% vs. 43%; *p* < 0.001), and to have a higher body mass index (31.4 vs. 30.5; *p* < 0.001). Those who developed AKI also had a significantly higher burden of comorbid conditions like atrial fibrillation (26% vs. 16%), coronary artery disease (34% vs. 22%), cancer (15% vs. 12%), congestive heart failure (CHF) (34% vs. 16%), CKD (38% vs. 17%), obstructive lung disease (21% vs. 17%), diabetes (42% vs. 26%), gastrointestinal bleed (11% vs. 8%), hypertension (66% vs. 50%), peripheral vascular disease (15% vs. 9 %) and respiratory failure (14% vs. 9%).

Patients who developed AKI were more likely to be on ACE/ARB (27% vs. 24%), antianginal medications (32% vs. 21%), anticoagulants (37% vs. 33%), diuretics (55% vs. 30%), lipid lowering medications (47% vs. 39 %) and nephrotoxic antibiotics (12% vs. 10%). The AKI group had higher values of blood urea nitrogen (BUN) (40.9 mg/dL vs. 19.2 mg/dL), serum creatinine (2.1 mg/dL vs. 1.0 mg/dL), and leukocyte count (11,190/mL vs. 9930/mL), and lower values of serum albumin (3.2 vs. 3.5 g/dL) and hemoglobin (10.6 vs. 11.5 g/dL). Using prespecified chronological cut-off, 192,720 (92.5%) admissions were assigned to the training set and 15,555 (7.5%) to the hold-out set, as shown in Supplemental Figure S1. The percentage of AKI in these datasets (12.5%) and well as their clinical characteristics, medications and lab values were similar as shown in Supplemental Table S2.

### 3.2. Model Development

In the training dataset, at 24 h of hospitalization, the top 15 predictors for developing AKI during that hospital stay are shown in Table 3. Many variables overlapped among the 3 different algorithms. The change in serum creatinine during the first 24 h of admission was the strongest predictor in all the models. Missing percentages for variables before imputation are shown in Supplemental Table S4. Of note, importance for Random Forest was defined by purity, the variance in the responses with the addition/subtraction of those predictors. For LASSO, importance was defined as the absolute value of coefficient of the corresponding predictor. For GBM, importance was defined based on the improvement made by each predictor in the split criterion across all the trees that the predictor was used. The relative importance of predictors is depicted in Supplemental Figure S2.

### 3.3. Model Performance

#### 3.3.1. ROC Curve and Precision-Recall Curve

**Figure 1:**
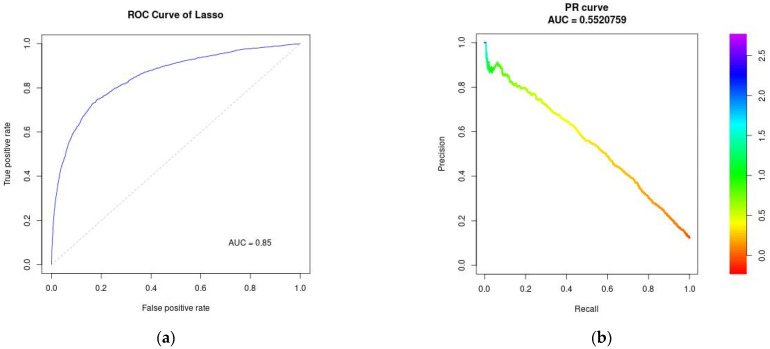
Performance evaluation for LASSO (**a**) Receiver Operating Characteristic (ROC) curve; (**b**) Precision-Recall (PR) curve. AUC: Area under the curve.

**Figure 2:**
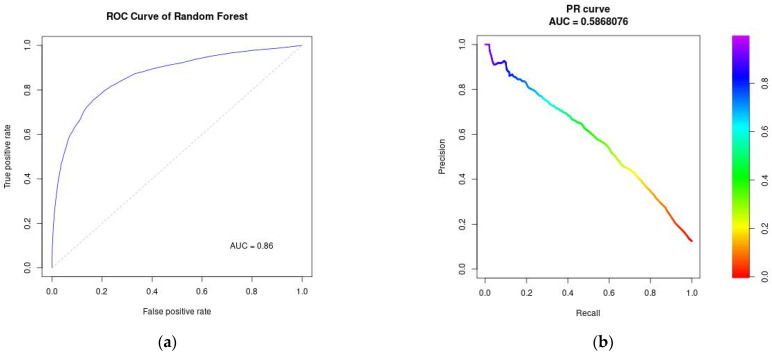
Performance evaluation for Random Forest (**a**) ROC curve; (**b**) Precision-Recall curve.

**Figure 3:**
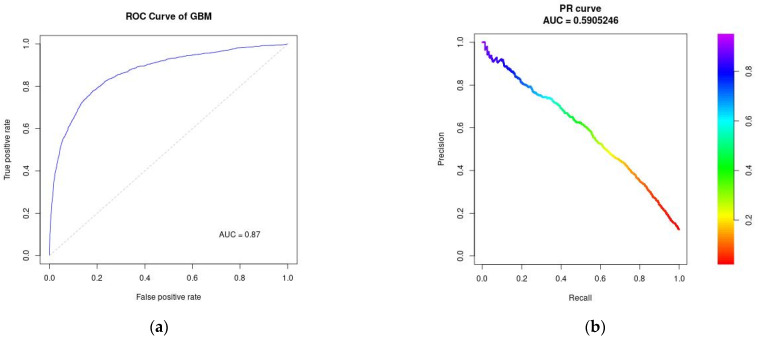
Performance evaluation for Gradient Boosting Machines (**a**) ROC curve; (**b**) Precision-Recall curve.

Discrimination performance is for each algorithm using AUROC and average precision is shown in Figure 1, Figure 2 and Figure 3. AUROC = 0.85 (95% CI: 0.84–0.86) for LASSO; AUROC = 0.86 (95% CI: 0.85–0.87) for Random Forest, and AUROC = 0.87 (95% CI: 0.86–0.88) for Gradient Boosting Machines. Average precision indicates Random Forest and Gradient Boosting Machines can correctly identify the positive HA-AKI without accidentally marking too many negative as positive (0.55 for LASSO; 0.59 for both Random Forest and Gradient Boosting Machines). Based on the Brier scores on the validation data (RF: 0.074; GBM: 0.075; Lasso: 0.076), all algorithms were well-calibrated.

#### 3.3.2. Performance Metrics by Threshold

Varying thresholds by algorithm showed specificity was closely linked to the thresholds, while sensitivity decreased as thresholds increased (Table 4). Youden Index was used to select the optimal threshold or cut-off in predicted probabilities. By varying the cut-off from list of 0.05 to 0.95 by 0.05 fixed interval, Youden threshold indicated that the optimal threshold fell between 0.15 and 0.20 across the 3 models. A cut-off at 0.15 yielded optimal balance between sensitivity and specificity for Random Forest.

#### 3.3.3. Time from Prediction to AKI and Performance in Dialysis Requiring AKI

The time interval between the model’s AKI prediction and actual occurrence of AKI was measured as a secondary outcome among the 1550 patients in the validation cohort where AKI was correctly predicted. Based on the selected Random Forest model with the Youden threshold of 0.15, the mean number of days from the time of prediction to the date of AKI were 2.3 with standard deviation of 2.7. The distribution of number of days where the model could predict AKI before it developed was positively skewed (skewness = 4.09) with median = 1 and interquartile range 1 to 3. Time between AKI prediction and occurrence was 1 day in 58.5%, 2 days in 14.9%, 3 days in and 9.1%, 4 days in 5.61%, 5 days in 3.4% and >5 days in 8.5% of patients who developed AKI.

A sensitivity analysis was also performed for model performance among the 104 patients in the validation cohort who required dialysis. The model correctly predicted AKI in 95 (90.5%) of those patients.

## 4. Discussion

Given the high prevalence and incremental costs associated with AKI, there has been great interest in use of AKI prediction models for early risk stratification. Various investigators have utilized traditional as well as machine learning prediction algorithms to achieve this. Unfortunately, widespread application of these models has been limited due to complex computational needs and variable performance [20,21]. To address limitations of prior models, we developed and validated a simpler model that can be operationalized using 15–20 variables obtained during the first 24 h of hospitalization only. Our model showed excellent prediction for AKI a mean of 2.3 days before it occurs and performed especially well with the patients who developed AKI requiring dialysis. Our Random Forest model had the best trade-off between sensitivity and specificity and achieved an AUROC of 0.86. All our models offered excellent negative predictive value, making it possible to stratify patients to lower risk for AKI within 24 h of hospital admission. We achieved a good balance between sensitivity and specificity for AKI prediction in validation; both around 80%. We also offer a single prediction model for all hospitalized patients rather than sub-populations.

Initial work on AKI prediction was limited by use of heterogenous AKI definitions, non-standardized methodology, and poor performance of traditional models [6]. An early machine learning predictive model used deep learning and a recurrent neural network to continuously predict AKI before 24, 48 and 72 h of its occurrence [22]. While they achieved an AUROC of 0.92 for AKI prediction within 48 h, the amount of data preprocessing required to integrate more than 600,000 input variables may prove to be prohibitive for clinical application. Moreover, widespread use would also be limited by the high proportion of false positive predictions [10]. Several subsequent machine learning AKI prediction models have been developed. Gameiro et al. reviewed most of these and found that many were limited to subsets of hospitalized patients like the elderly, cirrhotic, critically ill or those undergoing cardiac surgery, general surgery or coronary angiography. Hence, these models are limited in application, requiring use of multiple models and even more intense computational requirements to cover the entire spectrum of hospitalized patients [7].

On systematic review, eight AKI machine learning models were identified that have aimed to predict AKI in all medical and surgical admissions, an objective comparable to ours. While these strived to achieve excellence in AKI prediction, results have been heterogeneous. By design, most machine learning models are developed using retrospective datasets. Given their recent development, nearly all of them, including ours, lack external validation [7]. Of these, the model developed by Koyner et al. has been the most promising and subsequently has been externally validated. The continuously running model initially included 97 variables and was subsequently simplified to 59 variables. Hence, the computational requirements are expected to be significant if evaluating for widespread implementation. Moreover, the predictive performance is quite similar to our much simpler approach that requires fewer resources and only 15–20 variables [23]. The other machine learning models described showed similar or worse performance compared to our model, while still utilizing much more data points. In fact, our model’s sensitivity and specificity, both approaching 80%, is equal to or better than most prior reports. Hence, our study provides a potential pathway for simplification of AKI machine learning prediction models. Future studies are required to assess whether this approach can be simplified even further (fewer or simpler variables?) to reduce the data preprocessing and computational demands of these models.

Among a few validated prior models, Churpek et al. have developed and externally validated a gradient boosted machine AKI prediction model for predicting stage 2 AKI within a 48 h interval. While the model performs well, they excluded any patients with CKD stage 4 or those who required dialysis within 48 h of admission [23]. Further, while stage 1 AKI patients have better outcomes compared to stage 2 or 3, they still have much worse outcomes compared to patients with no AKI [1]. Our model addresses a wider hospitalized population and all stages of AKI. Prior attempts have been made to simplify prediction models using our approach: only using data gathered during the first 24 h of admission. One such group in the UK developed 3 Fuzzy Logic Systems models to identify any AKI, AKI stage 2/3 or AKI stage 3. Their AKI model, looking at an outcome similar to ours, had modest performance on internal validation, with AUROC 0.7 [11]. Another such attempt using the first 24 h of data by a group in the US led to machine AKI prediction models with only modest performance, an AUROC of 0.660–0.664 [24].

A prospective study of critically ill patients at admission found that machine learning models offer similar predictive ability as experienced clinicians but with less risk of overestimation [25]. As demonstrated by our model, it is possible to rapidly identify patients at lower risk for AKI during admission using data obtained during the first 24 h of admission, with negative predictive value in excess of 95%. Given the widespread prevalence of EHRs and our significant variables being clinical parameters routinely collected during the first 24 h of inpatient care, our model may prove to be easier to implement than existing models utilizing numerous parameters [26]. The standardized definitions of baseline creatinine also increase the probability that our model would be able to perform well across EHRs and healthcare systems. This can be contrasted with a recent personalized AKI prediction model where baseline creatinine was defined as values within or immediately prior to hospitalization. Moreover, that study also excluded even mild CKD patients (serum creatinine > 1.3 mg/dL), further limiting generalization to all hospitalized patients [27]. Interventions like implementation of guideline-based care bundles targeting patients at high risk for AKI, as defined by biomarkers, have been shown to reduce the frequency and severity of AKI after cardiac surgery [28]. Machine learning models like ours may allow similar risk stratification on broader scale among all hospitalized patients, with the hope of improving outcomes. One such approach is currently being tested in a National Institutes of Health–funded clinical trial [29].

Despite the ease of use and other strengths, there are several potential limitations to our algorithm. While our study used a standardized serum creatinine-based AKI definition based on KDIGO guidelines, we did not have urine output data available for all patients. Prior studies have shown that including urine output impacts estimates of prevalence and outcomes of AKI among hospitalized patients. However, almost all such studies have been exclusive to critically ill patients. Given that reliable urine output data is seldom available for the vast majority of hospitalized patients outside intensive care units, this remains an inherent limitation common in most AKI studies [30]. Our study focused on a single health system with a relatively homogenous population, and thus external validation is needed to ensure that our models do not suffer from hyperfit and are generalizable. It also remains to be seen whether risk stratification based on our model can actually improve patient outcomes. While a few preventative interventions, like management of hemodynamics and discontinuation of nephrotoxic medications seem reasonable, there remains the potential for patient harm by avoidance of clinically indicated contrast studies and inadequate medication dosing [10]. Prior studies have demonstrated the phenomenon of “renalism”: denial of medically indicated interventions to patients perceived to be at high risk for acute kidney injury [31]. This phenomenon will need to measured carefully in any clinical trials on our approach. Hence, the next logical step after external validation would be decision curve analyses (to assess impact in practice) and randomized trials of interventions based on the output of models like ours. 

## 5. Conclusions

We devised and internally validated a machine learning algorithm to accurately predict AKI 2.3 days before it occurs, using 20 common variables available within the first 24 h of hospitalization. The model performed well and requires external validation and evaluation of impact on patient outcomes.

## Figures and Tables

**Table 1:** Descending hierarchy to determine baseline creatinine. Rules in order of preference, 1 through 5.

Rule	Creatinine Available Prior to Admission	Baseline Creatinine
**1**	3 or more outpatient values available in 14–365 days prior to admission (PTA)	Mean of all outpatient values
**2**	2 outpatient values available in 14–365 days PTA + some prior inpatient values	Mean of 2 outpatient and lowest inpatient value
**3**	1 or fewer outpatient creatinine values in 14–365 days PTA but outpatient values available within 18 months PTA	Mean of all outpatient values
**4**	No outpatient creatinine values in 18 months PTA but patient had prior inpatient admissions	Mean of 3 lowest inpatient values
**5**	No prior inpatient or outpatient creatinine available	Creatinine at admission

**Table 2:** Characteristics of patients who developed AKI compared to those who did not.

	AKI	No AKI	*p* -Value
**Total, *n*, (%)**	**26,345 (12.6)**	**182,646 (87.4)**	
Age, mean (SD)	70.10 (15.5)	63.25 (19.0)	<0.001
Male gender, *n* (%)	13,746 (52.2)	79,178 (43.4)	<0.001
Body mass index, mean (SD)	31.38 (8.7)	30.51 (8.3)	<0.001
**Comorbidities**			
Atrial fibrillation, *n* (%)	6745 (25.6)	28,225 (15.5)	<0.001
Coronary artery disease, *n* (%)	8940 (33.9)	40,102 (22.0)	<0.001
Cancer, *n* (%)	3974 (15.1)	22,808 (12.5)	<0.001
Congestive Heart Failure, *n* (%)	9048 (34.3)	29,707 (16.3)	<0.001
Chronic Kidney Disease, *n* (%)	10,061 (38.2)	31,561 (17.3)	<0.001
Obstructive Lung Disease, *n* (%)	5645 (21.4)	31,688 (17.3)	<0.001
Diabetes Mellitus, *n* (%)	10,990 (41.7)	47,212 (25.8)	<0.001
Gastrointestinal Bleed, *n* (%)	3007 (11.4)	14,666 (8.0)	<0.001
Hypertension, *n* (%)	17,303 (65.7)	91,761 (50.2)	<0.001
Peripheral vascular disease, *n* (%)	4042 (15.3)	17,206 (9.4)	<0.001
Respiratory failure, *n* (%)	3749 (14.2)	16,746 (9.2)	<0.001
**Medications**			
ACE/ARB, *n* (%)	7177 (27.2)	44,061 (24.1)	<0.001
Antianginal medications, *n* (%)	8470 (32.2)	38,972 (21.3)	<0.001
Anticoagulants, *n* (%)	9692 (36.8)	59,381 (32.5)	<0.001
Diuretics, *n* (%)	14,345 (54.5)	54,681 (29.9)	<0.001
Lipid lowering medication, *n* (%)	12,470 (47.3)	71,550 (39.2)	<0.001
Nephrotoxic antibiotics, *n* (%)	3215 (12.2)	18,232 (10.0)	<0.001
**Lab measurements, mean (standard deviation)**			
Serum albumin, g/dL	3.21 (0.7)	3.54 (0.6)	<0.001
Total Bilirubin, mg/dL	1.2 (2.7)	0.8 (1.3)	<0.001
Blood urea nitrogen, mg/dL	40.9 (25.5)	19.2 (12.3)	<0.001
Serum creatinine, mg/dL	2.1 (1.4)	1.0 (0.5)	<0.001
Blood glucose, mg/dL	140 (63.3)	131 (53.6)	<0.001
Hemoglobin, g/dL	10.6 (2.1)	11.5 (2.1)	<0.001
Prothrombin time, INR	1.8 (1.1)	1.5 (0.8)	<0.001
Leukocyte count, ×1000/mL	11.2 (10.8)	9.9 (6.6)	<0.001

AKI: Acute Kidney Injury, ACE: Angiotensin Converting Enzyme Inhibitor, ARB: Angiotensin Receptor Blocker, INR: International Normalized Ratio.

**Table 3:** Top 15 variables in each algorithm.

LASSO	Random Forest	Gradient Boost
**Serum Creatinine** **	**Serum Creatinine** **	**Serum Creatinine** **
**CKD** **	eGFR *	eGFR *
**Diuretic use** **	Mean arterial pressure *	Mean arterial pressure *
Serum albumin	**CKD** **	**CKD** **
Calcium channel blocker	**Diuretic use** **	**Diuretic use** **
Vasodilator therapy	Body Mass Index *	White Blood Cell *
**Thrombocytopenia** **	Hypercalcemia *	Prothrombin time (INR) *
NSAID use	Hemoglobin *	Serum sodium
Steroid use	Platelet count *	Platelet count *
**Antidiabetic meds** **	White Blood Cell *	Hemoglobin *
Respiratory failure *	Prothrombin time (INR) *	**Antidiabetic meds** **
Serum potassium	**Thrombocytopenia** **	Body Mass Index *
Nephrotoxic antibiotics *	**Antidiabetic meds** **	**Thrombocytopenia** **
Cancer	Hypertension	Hypercalcemia *
Anticoagulants	Congestive Heart Failure	Respiratory failure *

* Variable represented in 2 algorithms. ** Variable represented in all 3 algorithms. CKD: Chronic Kidney Disease, eGFR: Estimated Glomerular Filtration Rate, NSAID: Nonsteroidal Anti-inflammatory Drugs, INR: International Normalized Ratio

**Table 4:** Sensitivity, specificity, and NPV by varying cut-off in Lasso, Random Forest and Gradient Boosting Machines.

LASSO			
Cut-Off	Sensitivity	Specificity	NPV
**0.05**	0.96	0.29	0.98
**0.10**	0.91	0.51	0.98
**0.15**	0.84	0.68	0.97
**0.20**	0.76	0.80	0.96
**0.50**	0.29	0.98	0.91
**Random Forest**			
**Cut-Off**	**Sensitivity**	**Specificity**	**NPV**
**0.05**	0.92	0.48	0.98
**0.10**	0.86	0.69	0.97
**0.15**	**0.80**	**0.79**	**0.96**
**0.20**	0.73	0.85	0.96
**0.50**	0.41	0.97	0.92
**Gradient Boosting Machines**			
**Cut-Off**	**Sensitivity**	**Specificity**	**NPV**
**0.05**	0.90	0.59	0.98
**0.10**	0.81	0.77	0.97
**0.15**	**0.74**	**0.85**	**0.96**
**0.20**	0.67	0.89	0.95
**0.50**	0.41	0.97	0.92

Metrics with cut-off probabilities above 0.50 are not listed as the sensitivity drops to a very low level. NPV: Negative Predictive Value.

## Data Availability

Data supporting reported results contains potentially identifiable patient information and hence cannot be shared to protect privacy.

## Supplementary Materials

The following supporting information can be downloaded at: https://www.mdpi.com/article/10.3390/jcm11195688/s1, Figure S1: CONSORT DIAGRAM; Figure S2: Top 15 features: relative importance in the 3 models; Table S1: Variables of interest considered for AKI prediction model; Table S2: Baseline and clinical characteristics in training and temporal validation cohort; Table S3: TRIPOD Checklist: Prediction Model Development; Table S4: Baseline and clinical characteristics in training and temporal validation cohort.
