# Supplementary material for: A Simpler Machine Learning Model for Acute Kidney Injury Risk Stratification in Hospitalized Patients
Source: J Clin Med. 2022 Sep 26;11(19):5688. doi: 10.3390/jcm11195688 (PMC9573390; doi:10.3390/jcm11195688)
Supplement: Supplementary file 1 [file jcm-11-05688-s001.zip › jcm-1849482-supplementary.pdf]

Table S1. Variables of interest considered for AKI prediction model

Type	Variables	Measured in prior models (n)
Demographic	Age	44
Demographic	Sex	42
Lab value	Serum creatinine closest to 24 hour mark	21
Demographic	Body mass index	20
Comorbid	Smoking	18
Comorbid	Hypertension	16
Comorbid	DM	15
Demographic	Race	15
Lab value	eGFR	14
Comorbid	Congestive heart failure	13
Comorbid	Diabetes	13
Comorbid	Hypertension	13
Comorbid	Left ventricular ejection fraction	13
Comorbid	Chronic obstructive pulmonary disease (COPD)	11
Comorbid	Peripheral vascular disease (PVD)	9
Surg detail	cardiopulmonary bypass duration	8
Comorbid	Cerebrovascular disease	8
Lab value	Creatinine	8
Lab value	Hemoglobin	8
Medication	Aminoglycoside	7
Comorbid	Mechanic Ventilation	7
Lab value	Platelet count	7
Lab value	Albumin	6
Lab value	Bilirubin	6
Comorbid	CKD	6
Medication	Diuretic use	6
Lab value	Potassium	6
Comorbid	Sepsis	6
Comorbid	Anemia	5
Comorbid	Atrial fibrilation	5
Lab value	BUN	5
Comorbid	Cardiogenic shock	5
Comorbid	Coronary artery disease	5
Comorbid	Diabetes mellitus	5
Demographic	Height	5
Comorbid	IABP use	5
Comorbid	Previous MI	5
Lab value	Sodium	5

Demographic	Weight	5
Lab value	Calcium	4
Comorbid	Cerebrovascular accident	4
Comorbid	Chronic Lung Disease	4
Surg detail	Cross clamp time	4
Comorbid	Hypotension	4
Medication	NSAIDS	4
Lab value	pH	4
Comorbid	Previous PCI	4
Lab value	proteinuria	4
Vital signs	Systolic BP	4
Vital signs	Temperature	4
Vital signs	urine output	4
Lab value	WBC count	4
Demographic	Weight	4
Lab value	eGFR	3
Lab value	ALT	3
Lab value	AST	3
Lab value	Bicarbonate	3
Comorbid	Chronic liver disease	3
Lab value	glucose	3
Vital signs	Heart rate	3
Lab value	Hematocrit	3
Comorbid	Hyperlipidemia	3
Comorbid	Liver disease	3
Surg detail	Operative time	3
Medication	pRBC transfusion	3
Medication	vasopressor agent use	3
Lab value	Prothrombin time	3
Comorbid	Stroke	3
Lab value	Total bilirubin	3
Medication	Vancomicyn	3
Medication	Calcium channel blocker	2
Medication	Inotropics	2
Medication	ACE inhibitors/ARB	2
Surg detail	aortic clamping duration	2
Medication	Beta-blocker use	2
Medication	Calcineurin inhibitor	2
Comorbid	Cancer	2
Surg detail	cardiopulmonary bypass (CPB)	2
Comorbid	Charlson score	2

Comorbid	Cirrhosis	2
Comorbid	CKD stage	2
Lab value	CRP	2
Comorbid	CV disease	2
Surg detail	CV surgery type (CABG;valve;other)	2
Comorbid	Ejection fraction	2
Comorbid	Endocarditis (yes/no)	2
Medication	Immunosuppressive treatment	2
Medication	IV Contrast	2
Vital signs	MAP	2
Lab value	MELD score	2
Comorbid	MI	2
Comorbid	NYHA CHF class	2
Medication	Other antibiotics	2
Lab value	Phosphorus	2
Comorbid	Previous cardiac surgery	2
Comorbid	prior CABG	2
Comorbid	prior cardiac surgery	2
Comorbid	prior valve surgery	2
Comorbid	Respiratory failure	2
Vital signs	Respiratory rate	2
Lab value	serum albumin	2
Lab value	serum sodium/Na	2
Vital signs	Severity of hypotension	2
Surg detail	Surgery type	2
Comorbid	Thrombocytopenia	2
Lab value	Troponin I	2
Surg detail	Urgency status of surgery: elective	2
Surg detail	urgent, emerg or emerg salvAge	2
Lab value	Uric acid	2
Lab value	WBC	2
Medication	Antidiabetic medications	New
Medication	steroids	New
Medication	Anticoagulants	New
Medication	Vasodilators	New
Medication	Lipid lowering drugs	New
Comorbid	Respiratory failure	New
Medication	Use of nephrotoxic antibiotic (vancomycin, aminoglycoside)	New
Medication	Antiplatelet agents	New

Figure S1. CONSORT DIAGRAM

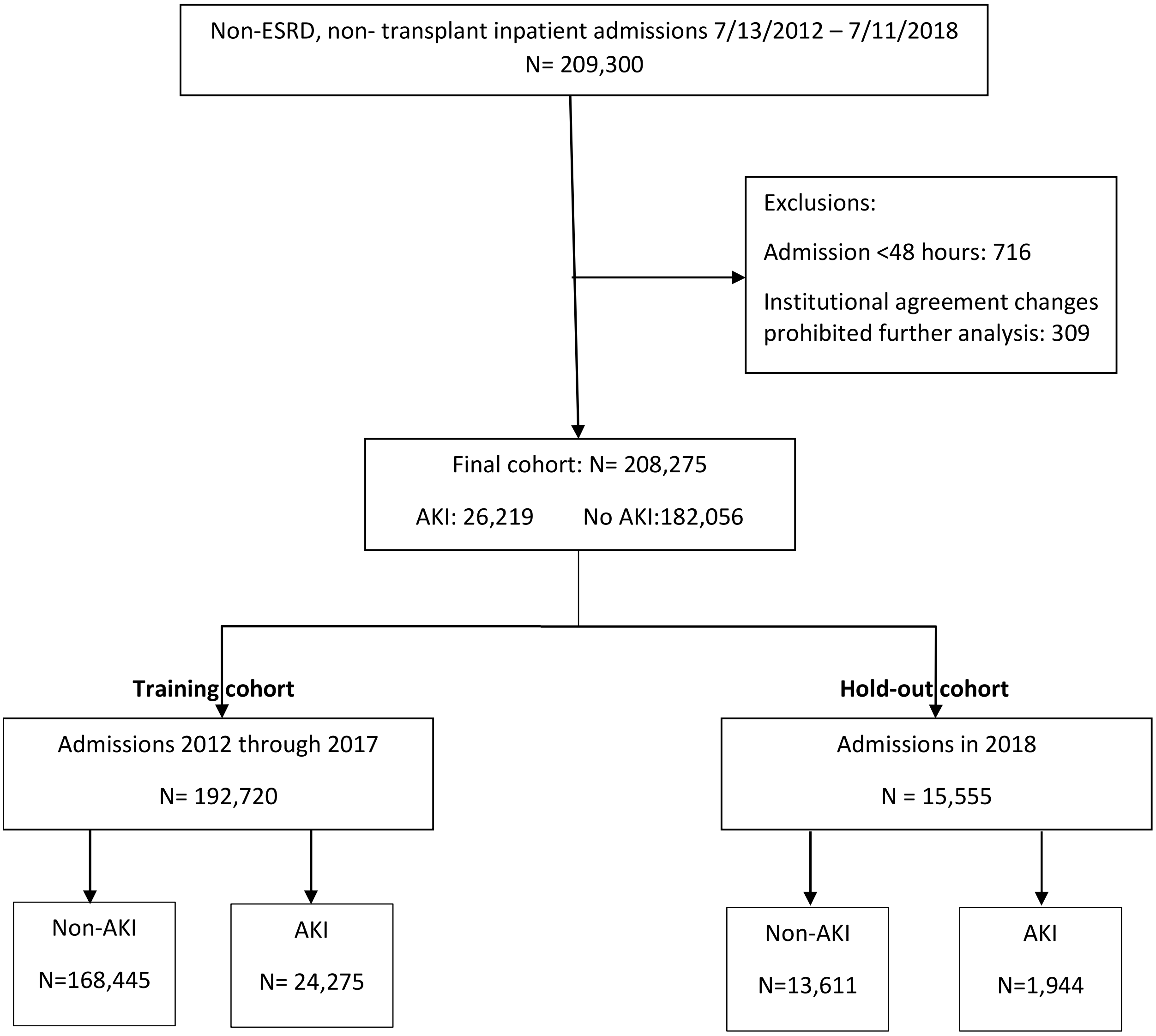

Table S2. Baseline and clinical characteristics in training and temporal validation cohort

	Training			Validation		
	**AKI**	**No AKI**	**p-value**	**AKI**	**NoAKI**	**p-value**
**Total, n (%)**	24275 (12.60)	168445 (87.40)		1944 (12.50)	13611 (87.50)	
**Age, mean (SD)**	70.07 (15.55)	63.19 (18.98)	<0.001	70.55 (15.25)	64.01 (18.92)	<0.001
**Sex, n (%)**	12658 (52.1)	73081 (43.4)	<0.001	1023 (52.6)	5825 (42.8)	<0.001
**BMI, mean (SD)**	31.37 (8.70)	30.49 (8.24)	0.004	31.43 (8.82)	30.77 (8.51)	<0.001
**BSA, mean (SD)**	1.97 (0.29)	1.93 (0.28)	<0.001	1.96 (0.30)	1.93 (0.29)	<0.001
**MAP, mean (SD)**	85.26 (15.40)	88.04 (12.78)	<0.001	85.30 (14.34)	87.92 (12.55)	<0.001
**Smoke Status, n (%)**			<0.001			<0.001
**Former Smoker**	11779 (48.5)	69105 (41.0)		933 (48.0)	5533 (40.7)	
**Never Smoker**	9224 (38.0)	65908 (39.1)		722 (37.1)	5466 (40.2)	
**Smoker**	3085 (12.7)	32496 (19.3)		274 (14.1)	2546 (18.7)	
**Comorbidities, n (%)**						
**Atrial fibrillation**	6364 (26.2)	26584 (15.8)	<0.001	364 (18.7)	1583 (11.6)	<0.001
**Arrhythmia**	3349 (13.8)	15934 (9.5)	<0.001	203 (10.4)	901 (6.6)	<0.001
**Asthma**	2111 (8.7)	18552 (11.0)	0.185	138 (7.1)	1088 (8.0)	<0.001
**Coronary artery disease**	8422 (34.7)	37906 (22.5)	<0.001	499 (25.7)	2102 (15.4)	<0.001
**Cancer**	3804 (15.7)	21900 (13.0)	0.004	160 (8.2)	877 (6.4)	<0.001
**Congestive Heart fAilure**	8542 (35.2)	27966 (16.6)	<0.001	483 (24.8)	1668 (12.3)	<0.001
**Chronic Kidney Disease**	9505 (39.2)	29860 (17.7)	<0.001	535 (27.5)	1633 (12.0)	<0.001
**Obstructive Lung Disease**	5306 (21.9)	29821 (17.7)	<0.001	323 (16.6)	1785 (13.1)	<0.001
**Diabetes Mellitus**	10318 (42.5)	44627 (26.5)	<0.001	637 (32.8)	2481 (18.2)	<0.001
**Gastrointestinal Bleed**	2845 (11.7)	13834 (8.2)	<0.001	156 (8.0)	802 (5.9)	<0.001
**Hypertension**	16341 (67.3)	86953 (51.6)	<0.001	911 (46.9)	4626 (34.0)	<0.001
**Hypercalcemia**	18664 (76.9)	127114 (75.5)	0.003	1501 (77.2)	10074 (74.0)	<0.001
**Myocardial infarction**	2426 (10.0)	9420 (5.6)	<0.001	138 (7.1)	603 (4.4)	<0.001
**Vascular disease**	3820 (15.7)	16212 (9.6)	<0.001	217 (11.2)	946 (7.0)	<0.001
**Recurrent urine infection**	4514 (18.6)	21393 (12.7)	<0.001	426 (21.9)	2081 (15.3)	<0.001

**Respiratory failure**	3502 (14.4)	15504 (9.2)	<0.001	225 (11.6)	1199 (8.8)	<0.001
**Thrombocytopenia**	17009 (70.1)	120633 (71.6)	0.745	1367 (70.3)	9518 (69.9)	<0.001
**Heart Valve disease**	3207 (13.2)	14025 (8.3)	<0.001	180 (9.3)	748 (5.5)	<0.001
**Angina**	7824 (32.2)	35918 (21.3)	<0.001	622 (32.0)	2932 (21.5)	<0.001
**Medication, n (%)**						
**ACE inhibitor/ Angiotensin Blocker**	6611 (27.2)	40611 (24.1)	0.003	535 (27.5)	3317 (24.4)	<0.001
**Anticoagulants**	8887 (36.6)	54878 (32.6)	<0.001	756 (38.9)	4310 (31.7)	<0.001
**Antidiabetic medication**	10497 (43.2)	45444 (27.0)	<0.001	883 (45.4)	3814 (28.0)	<0.001
**Beta Blocker**	14425 (59.4)	74341 (44.1)	<0.001	1114 (57.3)	6048 (44.4)	<0.001
**Calcium Channel Blocker**	6313 (26.0)	30547 (18.1)	<0.001	574 (29.5)	2943 (21.6)	<0.001
**Diuretic**	13198 (54.4)	50297 (29.9)	<0.001	1079 (55.5)	4210 (30.9)	<0.001
**Lipd Lowering Drugs**	11475 (47.3)	65743 (39.0)	<0.001	940 (48.4)	5569 (40.9)	<0.001
**Nephrotoxic Antibiotic**	2943 (12.1)	16436 (9.8)	0.712	250 (12.9)	1706 (12.5)	<0.001
**Nonsteroidal analgesics**	3225 (13.3)	43978 (26.1)	<0.001	288 (14.8)	3699 (27.2)	<0.001
**Steroid**	4528 (18.7)	32889 (19.5)	0.887	421 (21.7)	2971 (21.8)	0.001
**Vasodilator**	2672 (11.0)	9971 (5.9)	<0.001	225 (11.6)	1041 (7.6)	<0.001
**Lab Measurements within 24-Hour, mean (SD)**						
**Serum Albumin, g/dL**	3.22 (0.65)	3.55 (0.61)	<0.001	3.20 (0.62)	3.51 (0.59)	<0.001
**Total Bilirubin mg/dL**	1.23 (2.70)	0.80 (1.36)	<0.001	1.06 (2.66)	0.69 (1.10)	<0.001
**Blood Urea Nitrogen, mg/dL**	40.79 (25.35)	19.16 (12.19)	<0.001	42.45 (26.91)	19.54 (12.91)	<0.001
**Serm Calcium, mg/dL**	8.56 (0.82)	8.70 (0.67)	<0.001	8.52 (0.77)	8.67 (0.65)	<0.001
**Serum Creatinine, mg/dL**	2.09 (1.35)	0.98 (0.45)	<0.001	2.10 (1.36)	0.98 (0.45)	<0.001
**Glucose,mg/dL**	139.75 (63.14)	130.89 (53.31)	<0.001	143.20 (65.01)	134.16 (56.69)	<0.001
**Hemoglobin, g/dL**	10.57 (2.10)	11.48 (2.07)	<0.001	10.47 (2.17)	11.44 (2.10)	<0.001
**Platelet 1000/mL**	202.35 (100.04)	216.90 (94.00)	<0.001	210.67 (104.39)	221.41 (94.75)	<0.001
**Potassium, meq/L**	4.27 (0.66)	4.07 (0.50)	<0.001	4.31 (0.69)	4.09 (0.50)	<0.001
**PT/INR**	1.75 (1.07)	1.47 (0.77)	<0.001	1.68 (0.96)	1.41 (0.72)	<0.001
**Serum Sodium, meq/L**	137.96 (5.14)	138.22 (4.03)	0.241	138.78 (5.15)	138.90 (4.05)	<0.001

**Leukocytes, 1000/mL**	11.23 (10.98)	9.94 (6.61)	<0.001	10.70 (7.98)	9.83 (5.38)	<0.001
**Estimated GFR, mL/min/1.73m^2**	40.99 (27.07)	75.46 (28.81)	<0.001	40.24 (26.63)	74.47 (28.52)	<0.001

Figure S2. Top 15 features: relative importance in the 3 models

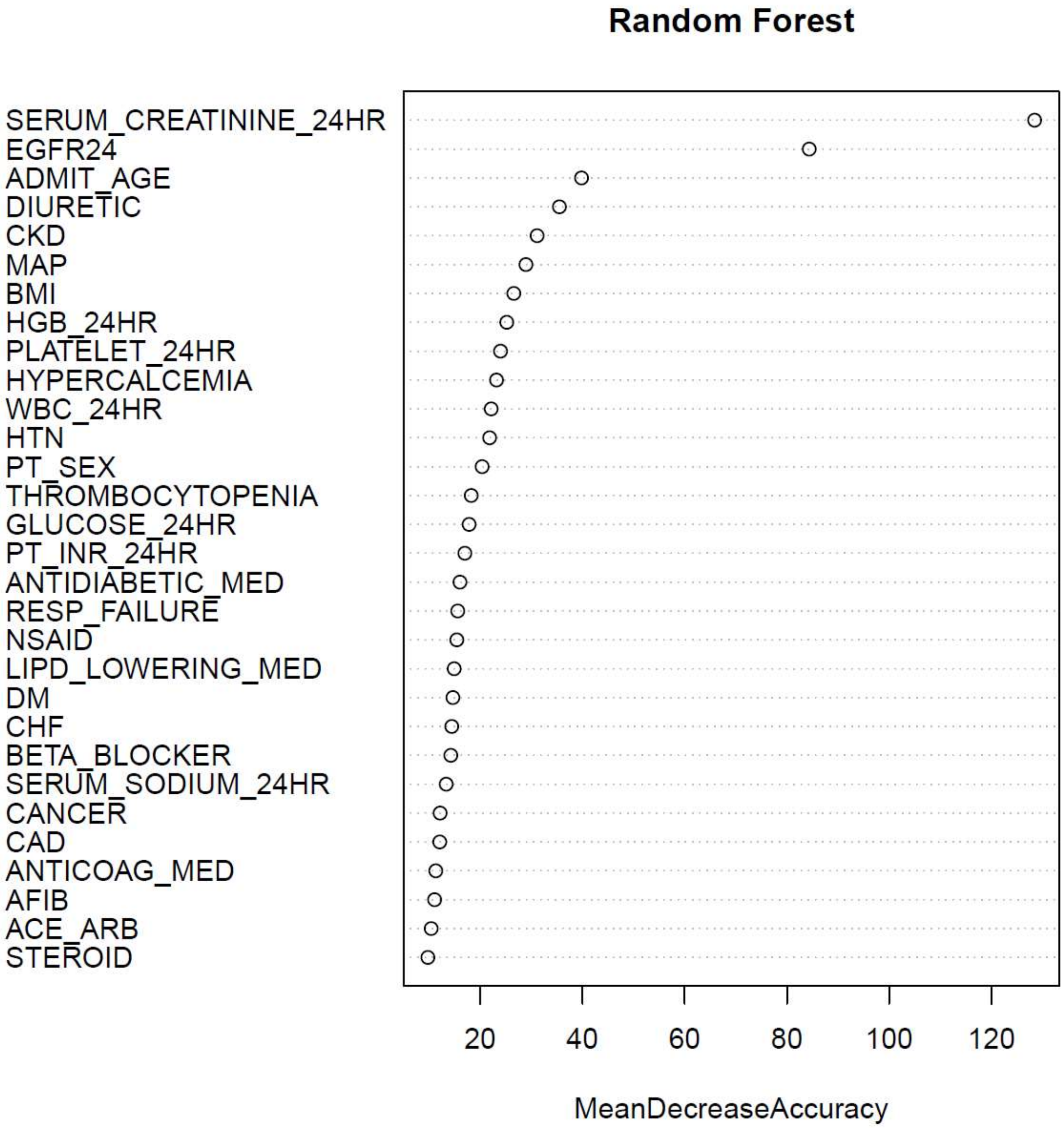

Gradient Boosted Model

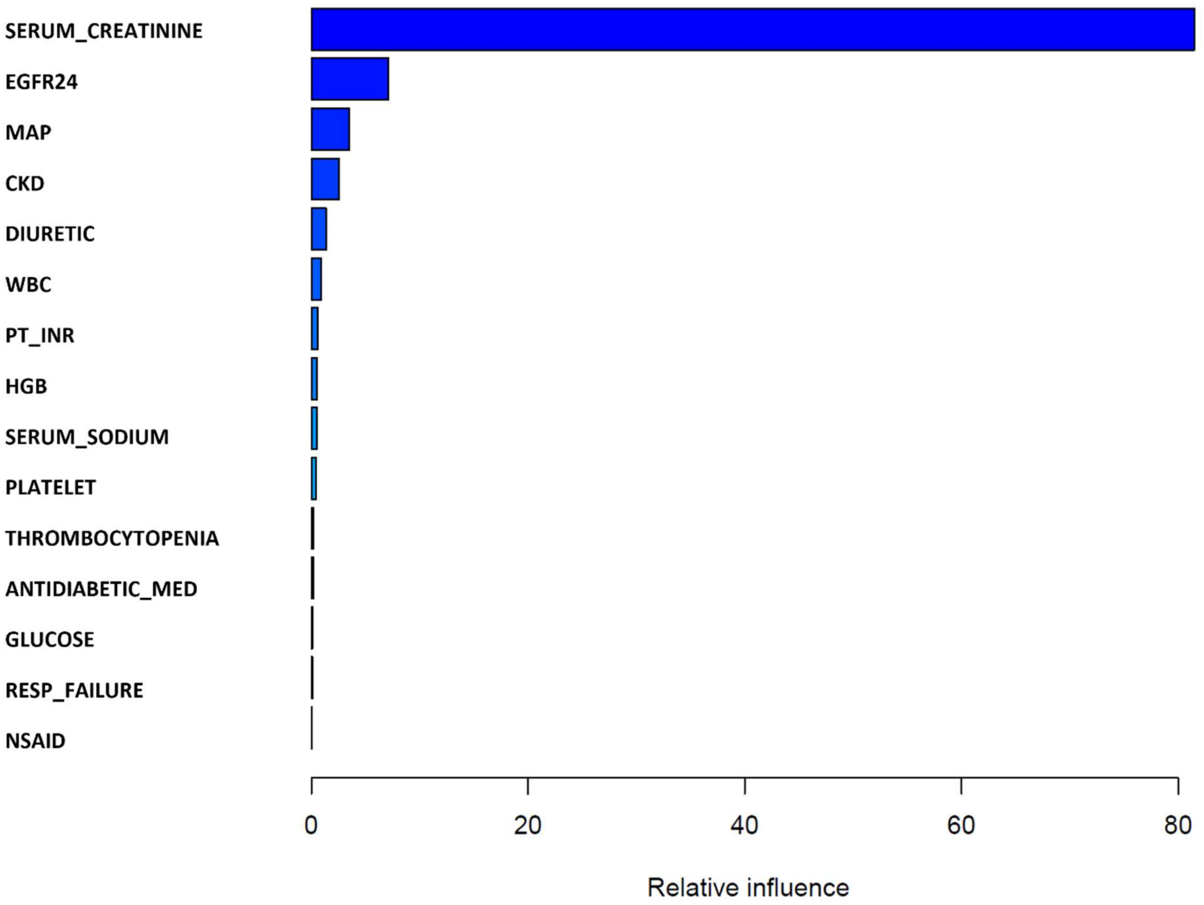

Top 15 features and coefficient from LASSO regression

	Coefficient	OR
Serum creatinine	0.68	1.98
eGFR	-0.64	0.53
CKD	-0.59	0.55
Diuretic use	0.41	1.50
BUN	0.31	1.37
NSAID use	-0.21	0.81
Age	-0.20	0.82
Mean Arterial Pressure	-0.18	0.83

Nephrotoxic antibiotics	0.18	1.19
Hypercalcemia	-0.17	0.85
Calcium blocker	0.16	1.17
Thrombocytopenia	-0.16	0.85
Steroid use	-0.16	0.86
Serum albumin	-0.15	0.86
Cancer	0.14	1.15

Table S3. TRIPOD Checklist: Prediction Model Development

Section/Topic		Checklist Item	Page
**Title and abstract**			
Title	1	Identify the study as developing and/or validating a multivariable prediction model, the target population, and the outcome to be predicted.	1
Abstract	2	Provide a summary of objectives, study design, setting, participants, sample size, predictors, outcome, statistical analysis, results, and conclusions.	1
**Introduction**			
Background and objectives	3a	Explain the medical context (including whether diagnostic or prognostic) and rationale for developing or validating the multivariable prediction model, including references to existing models.	1-2
	3b	Specify the objectives, including whether the study describes the development or validation of the model or both.	2-4
**Methods**			
Source of data	4a	Describe the study design or source of data (e.g., randomized trial, cohort, or registry data), separately for the development and validation data sets, if applicable.	2
	4b	Specify the key study dates, including start of accrual; end of accrual; and, if applicable, end of follow-up.	2
Participants	5a	Specify key elements of the study setting (e.g., primary care, secondary care, general population) including number and location of centres.	2
	5b	Describe eligibility criteria for participants.	2
	5c	Give details of treatments received, if relevant.	N/A
Outcome	6a	Clearly define the outcome that is predicted by the prediction model, including how and when assessed.	2-3
	6b	Report any actions to blind assessment of the outcome to be predicted.	N/A
Predictors	7a	Clearly define all predictors used in developing or validating the multivariable prediction model, including how and when they were measured.	3-4
	7b	Report any actions to blind assessment of predictors for the outcome and other predictors.	N/A
Sample size	8	Explain how the study size was arrived at.	4
Missing data	9	Describe how missing data were handled (e.g., complete-case analysis, single imputation, multiple imputation) with details of any imputation method.	3-4
Statistical analysis methods	10a	Describe how predictors were handled in the analyses.	3-4
	10b	Specify type of model, all model-building procedures (including any predictor selection), and method for internal validation.	4
	10d	Specify all measures used to assess model performance and, if relevant, to compare multiple models.	4
Risk groups	11	Provide details on how risk groups were created, if done.	N/A
**Results**			
Participants	13a	Describe the flow of participants through the study, including the number of participants with and without the outcome and, if applicable, a summary of the follow-up time. A diagram may be helpful.	4-5
	13b	Describe the characteristics of the participants (basic demographics, clinical features, available predictors), including the number of participants with missing data for predictors and outcome.	5-6
Model development	14a	Specify the number of participants and outcome events in each analysis.	4-5
	14b	If done, report the unadjusted association between each candidate predictor and outcome.	Supp
Model specification	15a	Present the full prediction model to allow predictions for individuals (i.e., all regression coefficients, and model intercept or baseline survival at a given time point).	6-8
	15b	Explain how to the use the prediction model.	8-9
Model performance	16	Report performance measures (with CIs) for the prediction model.	6-7
**Discussion**			
Limitations	18	Discuss any limitations of the study (such as nonrepresentative sample, few events per predictor, missing data).	9

Interpretation	19b	Give an overall interpretation of the results, considering objectives, limitations, and results from similar studies, and other relevant evidence.	9-10
Implications	20	Discuss the potential clinical use of the model and implications for future research.	9-10
**Other information**			
Supplementary information	21	Provide information about the availability of supplementary resources, such as study protocol, Web calculator, and data sets.	10
Funding	22	Give the source of funding and the role of the funders for the present study.	11

Table S4. Baseline and clinical characteristics in training and temporal validation cohort

Feature	Missing %
**Body mass index**	19.00%
**Body Surface Area**	19.00%
**Mean Arterial Pressure**	0.42%
**Serum albumin**	47.68%
**Total Bilirubin**	49.13%
**Blood Urea Nitrogen**	13.31%
**Serum Calcium**	13.26%
**Serum creatinine**	16.32%
**Blood Glucose**	12.96%
**Hemoglobin**	8.50%
**Platelet count**	10.67%
**Serum Potassium**	12.59%
**Prothrombin time**	46.10%
**Serum sodium**	12.89%
**White Blood Cell**	10.69%
**eGFR**	16.32%
